# Sodium butyrate alleviates R97-116 peptide-induced myasthenia gravis in mice by improving the gut microbiota and modulating immune response

**DOI:** 10.1186/s12950-023-00363-w

**Published:** 2023-11-03

**Authors:** Jing Sun, Juanjuan Chen, Qinfang Xie, Mengjiao Sun, Wenjing Zhang, Hongxia Wang, Ning Liu, Qi Wang, Manxia Wang

**Affiliations:** 1 Department of Neurology, Lanzhou University Second Hospital, Lanzhou, 730030 China; 2 Cuiying Biomedical Research Center, Lanzhou University Second Hospital, Lanzhou, 730030 Gansu China; 3 Department of Neurology, Qinghai Provincial People’s Hospital, Xining, 810007 China

**Keywords:** Myasthenia gravis, Gut microbiota, Sodium butyrate, Immune response, Differentially expressed genes of B cells

## Abstract

Fermented butyrate exhibits an anti-inflammatory response to maintain immune homeostasis within the gut. However, the effect and underlying mechanism of butyrate on myasthenia gravis (MG) remain unclear. The changes in the gut microbiota and fecal contents of SCFAs in MG patients were examined. R97-116 peptide was used to induce the experimental autoimmune myasthenia gravis (EAMG) mice and sodium butyrate (NaB) was gavaged to the EAMG mice. Gut microbiota, the frequency of Th1, Th17, Treg, Tfh, and B cells, the levels of IFN-γ, IL-17 A, IL-10, IL-21, and anti-R97-116 IgG, RNA-seq of total B cells in the spleen were explored by metagenomics, flow cytometry, ELISA, and transcriptomics. A significant reduction in SCFA-producing bacteria including Butyricimonas synergistica and functional modules including butyrate synthesis/production II was observed in MG patients and fecal SCFAs detection confirmed the increase. The EAMG mice were successfully constructed and NaB supplementation has changed the composition and function of the gut microbiota. The numbers of Th1, Th17, Tfh, and B cells were significantly increased while that of Treg cells was obviously decreased in EAMG mice compared with controls. Interestingly, NaB treatment has reduced the amounts of Th17, Tfh, and B cells but increased that of Treg cells. Accordingly, the levels of IL-17 A, IL-21, and IgG were increased while IL-10 was decreased in EAMG mice. However, NaB treatment reduced IL-17 A and IL-21 but increased that of IL-10. RNA-seq of B cells has revealed 4577 deferentially expressed genes (DEGs), in which 1218 DEGs were up-regulated while 3359 DEGs were down-regulated in NaB-treated EAMG mice. GO enrichment and KEGG pathway analysis unveiled that the function of these DEGs was mainly focused on immunoglobulin production, mitochondrial respiratory chain complex, ribosome, oxidative phosphorylation, and CNS diseases including amyotrophic lateral sclerosis. We have found that butyrate was significantly reduced in MG patients and NaB gavage could evidently improve MG symptoms in EAMG mice by changing the gut microbiota, regulating the immune response, and altering the gene expression and function of B cells, suggesting NaB might be a potential immunomodulatory supplement for MG drugs.

**Supplementary Information:**

The online version contains supplementary material available at 10.1186/s12950-023-00363-w.

## Introduction

Myasthenia gravis (MG) is an exemplary autoimmune disease that is dependent on CD4 + T cells and mediated by B cells. It is characterized by muscle weakness and rapid fatigue [1]. The underlying mechanism of MG primarily involves the production of autoantibodies that target the acetylcholine receptor (AChR) in 85% of patients [2], However, some patients exhibit autoantibodies against muscle-specific tyrosine kinase (MuSK) and low-density lipoprotein receptor-related protein 4 (LRP4). Furthermore, a smaller subgroup of patients present autoantibodies against various other postsynaptic proteins [3].

CD4 + T cells and cytokines play crucial roles in the pathogenesis of MG [4]. In vitro studies have revealed elevated proportions of T helper 1 (Th1) and T helper 17 (Th17) cells, derived from CD4 + T cells, in the peripheral blood mononuclear cells of MG patients, suggesting the potential pathogenicity of Th1 and Th17 cells in MG [5]. Furthermore, the autocrine factor IFN-γ, produced by Th1 cells, has been strongly associated with MG [6]. Additionally, the presence of IL-17, secreted by Th17 cells, has been implicated in the pathogenesis of various inflammatory and autoimmune diseases [7], underscoring its significance in the evaluation of MG. T regulatory cells (Tregs) are believed to have a crucial role in preserving peripheral immune tolerance against self-antigens. This is achieved through the utilization of various soluble mediators, such as IL-10. It is postulated that Tregs function by suppressing the effector CD4 + T cell subsets responsible for initiating autoimmune responses [8]. Besides, the imbalance of Th17 and Tregs was also reported as a pathological mechanism of MG [9]. Interestingly, recently studies have showed that the frequencies of follicullar helper T (Tfh) cells are significantly higher in MG patients compared with healthy controls, and Tfh cells are positively correlated with levels of serum anti-AChR antibody, and Tfh-Th17 cells may play a role in the immunopathogenesis of MG [10]. Moreover, it was observed that Tfh cells obtained from patients with MG stimulated B cells to generate antibodies through a mechanism that relies on IL-21 signaling [11]. The above studies indicate that the dysregulation of these four T helper cell subsets is involved in the pathogenesis of MG, thereby suggesting that modulating the differentiation of these T helper cells could potentially be employed as a therapeutic strategy for the management of MG.

As an archetypal autoimmune disease, the pathology of MG is involved in changes in autoantibodies secreted by B cells. Recent studies have reported that B cell-targeting monoclonal antibody (mAb) therapies for MG are increasingly attractive due to their specificity and efficacy [12]. However, the investigation of pathogenic B cells and antibodies derived from MG patients has been limited, primarily due to the challenges associated with their isolation [13], not to mention the gene expression of B cells in MG with and without treatment. Collectively, the numbers of Th1, Th17, Treg, Tfh, and B cells and contents of IFN-γ, IL-17, IL-10, IL-21, and titer of IgG could be useful indicators for the treatment of MG.

Gut microbiota has exerted a considerable influence on human neurophysiology and mental health [14]. Recently, some studies have demonstrated the relationship between gut microbiota dysbiosis and MG, and it is hypothesized that perturbation of the gut microbiota is associated with the pathogenesis of MG [15]. Totzeck et al. have revealed that Deltaproteobacteria and Faecalibacterium were abundant within the fecal microbiota of MG patients compared with controls [16]. Liu et al. have built an MG disease classifier based on the abundance of five species, *Fusobacterium mortiferum*, *Prevotella stercorea*, *Prevotella copri*, *Megamonas funiformis*, and *Megamonas hypermegale*, and the microbial markers might serve as novel diagnostic methods for pediatric MG [17]. Qiu et al. have demonstrated that the gut microbiota of the MG group was changed in terms of the relative abundances of bacterial taxa, with sharply reduced microbial richness, particularly in the genus *Clostridium* compared to the healthy cohort, and the fecal short-chain fatty acids (SCFAs) content was significantly lower in the MG group [18]. SCFA, specifically butyrate, plays an important role in mediating the effects of the gut microbiome on local and systemic immunity [19]. M D Säemann et al. has demonstrated that butyrate could exert anti-inflammatory effects by inhibiting IL-12 and up-regulating IL-10 in human monocytes [20]. J-P Segain et al. reported that butyrate could decrease pro-inflammatory cytokine expression via inhibition of NF-κB activation and IκBα degradation [21]. He et al. demonstrated that butyrate from bacterial metabolism could enhance the CD8 + T cell response and improve chemotherapy efficacy through ID2-dependent IL-12 signaling [22]. William Yip et al. concluded that butyrate could shape cell fate and function in allergic asthma [23]. These results suggested that butyrate plays an important role in anti-inflammtory and immune response, which might alleviate the symptoms of MG, an autoimmune disease.

In this study, we first explored the composition and function of gut microbiota in MG patients and revealed a significant reduction in the microbial metabolite butyrate. Based on that, an experimental autoimmune MG model (EAMG) in mice was constructed, and NaB was gavaged to the EAMG mice for 6 weeks to explore the effects of butyrate on MG and the underlying mechanism. Our study will provide a theoretical basis for novel treatment of MG in clinical.

## Methods and materials

### Human cohort information

MG patients were diagnosed according to previous studies [24, 25], and the Quantitative Myasthenia Gravis scale (QMG) test [26] and Hamilton Anxiety Scale (HAMA) [27] were used to evaluate the severity and mental status of MG. Participants with an extreme diet (e.g., vegan), a known history of inflammatory diseases such as inflammatory bowel disease or severe cardiac, hepatic, or renal diseases, received any antibiotics in one month or took probiotics, prebiotics, or glucocorticoids within three months were all excluded. Totally 18 patients with MG (average age 48.75 ± 15.88; BMI 23.13 ± 4.84; sex, male: female, 11:7) and 16 healthy controls (average age 48.75 ± 15.88; BMI 20.82 ± 1.36; sex, male: female, 10:6) were recruited from the Neurology Department and Physical Examination Center of Lanzhou University Second Hospital (Table S1). 5-10 g stool samples from MG patients and HCs were collected into a 2 mL labeled sterile tube and transported to the laboratory with dry ice to perform DNA extraction and SCFAs detection.

### Animal experiments

Specific pathogen-free C57BL/6J mice (female, 6 weeks) were purchased from Vital River Laboratories (Beijing, China). All mice were housed on a 12/12 light-dark schedule with free water and food.

R97-116 peptide (DGDFAIVKFTKVLLDYTGHI) was purchased from CSBio CO. LTD. (California, USA) to induce the experimental autoimmune MG (EAMG) mice model according to the published protocols [28]. Specifically speaking, 50 µg R97-116 peptide antigens were emulsified in Complete Freund’s Adjuvant (CFA; Sigma, St Louis, MO) and supplemented with additional nonviable Mycobacterium tuberculosis H37RA 1 mg/mice (Difco Laboratories, Detroit, MI). On day 0, 200 µL of the emulsion was subcutaneously injected into both hind footpads of the MG mice, and the controls were injected with CFA. On days 28 and 56, the mice were immunized again with R97-116 peptide solution at the tail base and back, respectively. Mice were monitored on alternate days for body weight, and the clinical score was recorded by a double-blind evaluation as previously described [28, 29].

NaB was purchased from Sigma-Aldrich and was dissolved in saline. From day 28 to 70, the MG mice were gavaged with NaB (200 mg/kg/d) once a day for 6 weeks. The control mice were given an equal volume of saline.

The mice were sacrificed for sampling as long as the experiment was finished. Fecal samples were collected for shotgun metagenomic sequencing. Blood was collected for IFN-γ, IL-17, IL-10, IL-21, and IgG detection. Spleens were collected to examine the frequency of Th1, Th17, Treg, Tfh, and B cells, and the RNA-seq of B cells. Inguinal lymph nodes were collected for examination of Th1, Th17, and Treg cells. The heart, liver, spleen, lung, kidney, colon, and stomach were collected for histopathological examination.

### Flow cytometry detection for Th1, Th17, Treg, Tfh, and B cells

As previously described, the excised spleen tissues and inguinal lymph nodes were fractionated to obtain a single-cell suspension [30–33]. Erythrocytes in spleens were lysed using Red Cell Lysis Buffer (Sigma-Aldrich). For intracellular staining, single-cell suspension was stimulated with phorbol myristic acetate (50 ng/mL), ionomycin (1 µg/mL), and monensin (2 µg/mL) (Sigma-Aldrich, St. Louis, MO, USA) for 4 h at 37 °C, then fixed and permeabilized with the buffers of a Foxp3 detection kit (eBioscience). Cell surface proteins were stained with FITC-anti-CD4 for 20 min at 4 °C in FACS buffer (PBS, 1 mM EDTA, 0.1% azide, and 1% BSA). Intracellular cytokines were stained with PE-anti-IFN-γ antibody for Th1 cells, APC-anti-IL-17 antibody for Th17 cells, and APC-anti-Foxp3 antibody for Tregs for 1 h at room temperature. For Tfh, cells were labeled with FITC-anti-CD4, APC-anti-CXCR5, and PE-anti-PD-1. For B, cells were labeled with APC-anti-B220 and FITC-anti-IgM. Stained cells were detected by flow cytometry (BD) and analyzed by FlowJo V10.8.1 software (Tree Star, Inc. San Carlos, CA, USA). All flow antibodies were purchased from Biolegend.

### Enzyme linked immunosorbent assay (ELISA) for cytokines and IgG

The levels of IFN-γ, IL-17 A, IL-10, and IL-21 in serum were measured by using a commercially available ELISA kit (NeoBioscience, China) according to the manufacturer’s instructions.

To evaluate anti-R97-116-specific IgG antibody production, 96-well microtiter plates (Thermo Fisher Scientific, USA) were coated with 10 µg/mL of peptide R97-116. Plates were incubated with serum samples in a ratio of 1:100, and the binding antibodies were detected by using biotin goat anti-mouse IgG (NeoBioscience, China).

### Histological analysis

The tissue from the heart, liver, spleen, lung, kidney, colon, and stomach was fixed in 4% formalin and embedded in paraffin. The paraffin-embedded tissues were cut into 5 mm thick pieces and stained with Hematoxylin and Eosin (H&E) for anatomical pathology diagnosis.

### Shotgun metagenomic sequencing of fecal samples

#### DNA extraction and sequencing

Stool DNA was extracted per the MetaHIT protocol described previously [34]. The DNA concentration was estimated by Qubit (Invitrogen). Library preparation was prepared as previously reported [35]. Metagenomic shotgun sequencing was performed on the BGI-SEQ500 platform according to the BGISEQ-500 protocol (SOP AO) employing the PE150 (paired-end library of 350-bp and 150-bp read length) mode as described [35].

Raw reads with FASTQ format that had 50% low-quality bases (quality ≤ 20; default parameters) or more than five ambiguous bases were excluded by fastp (https://github.com/OpenGene/fastp). The remaining reads were mapped to the human genome (Hg38) and mouse genome (GRCm39, https://asia.ensembl.org/Mus_musculus/Info/Index) by bowtie2 (https://bowtie-bio.sourceforge.net/bowtie2/index.shtml; default parameters) to remove host DNAs. High-quality nonhuman and non-mouse reads were defined as clean reads.

#### Taxonomic and functional annotation

The clean reads were used to produce taxonomic profiles for cohort and mice using MetaPhlAn3 [36] (- input_type fastq, - ignore_viruses, - nproc 6). The functional profile including gut metabolic modules (GMMs) (-a 2 -d GMM.v1.07.txt -s average) reported by Vieira-Silva et al. [37] and gut-brain modules (GBMs) reported by Valles-Colomer et al. [38] was calculated by HUMAnN3, a method for efficiently and accurately profiling the abundance of microbial metabolic pathways and other molecular functions from metagenomic or metatranscriptomic sequencing data developed by Francesco Beghini et al. [36].

#### Permutational multivariate analysis of variance

Permutational Multivariate Analysis of Variance (PERMANOVA; code: R 4.0.3: adonis (dist ~ phe, permutations = 9999) was performed based on the gut taxonomic profiles and functional modules to study the effect of clinical indexes on the gut microbiome.

#### Diversity

Alpha-diversity (within-sample diversity, R 4.0.3: diversity (data, index = ‘Shannon’)) and beta-diversity (between-sample diversity, R 4.0.3: pcoa (dis. bray, correction="none,“ rn = NULL) was calculated using the Shannon index and bray distance depending on the species profile and functional modules.

### Fecal SCFAs detection

SCFAs were measured by gas chromatography/mass spectrometry (GC/MS) as described by Zhang et al. [39]. Briefly, SCFAs were extracted with anhydrous ether from acidified fecal water extract, followed by dehydration with sodium sulfate and N, O-bis (trimethyl-silyl)-trifluoroacetamide derivatization at a reduced temperature. Selection monitoring mode was used for highly sensitive quantification of SCFAs by GC/MS. Major SCFAs including acetic acid, propionic acid, isobutyric acid, butyric acid, isovaleric acid, and valeric acid were identified and quantified accurately.

### RNA sequencing of B cells

#### RNA extraction and sequencing

Total B cells in the spleen were isolated and stored in Trizol to extract RNA. Total RNA in B cells was extracted by using the Trizol Reagent (Invitrogen Life Technologies). The concentration, quality, and integrity were determined by using a NanoDrop spectrophotometer (Thermo Scientific). Only samples with RNA integrity number (RIN) ≥ 7.0 were used to generate transcriptome libraries. In our study, mRNA-enriched transcriptome libraries were constructed. Enriched mRNA was used for transcriptome library construction using the TruSeq RNA Library Prep Kit v2 (Illumina), with an insert size of 380 bp. The sequencing library was then pair-end (PE150) sequenced on NovaSeq 6000 platform (Illumina) by Shanghai Personal Biotechnology Co. Ltd.

#### Quality control and reads mapping

Raw data in FASTQ format were generated by the sequencing platform. Cutadapt (v1.15) software was used to filter a number of connectors and low-quality reads to get high-quality sequences (clean data) for further analysis. Non-rDNA/rRNA reads were then mapped to the mouse genome (GRCm39) using Tophat2 (version 2.0.9, default parameters) as described by Kim D et al. [40] to remove potential host DNA and RNA contaminations. The reference genome and gene annotation files were downloaded from the genome website. The filtered reads were mapped to the reference genome using HISAT2 v2.0.5 (HISAT2 (daehwankimlab.github.io), default parameters).

#### Differential expression analysis

We used HTSeq (0.9.1) statistics to compare the Read Count values on each gene as the original gene expression and then used FPKM to standardize the expression. Then the differential expression of genes was analyzed by DESeq (1.30.0) with screened conditions as follows: expression differences multiple |log2 (Fold Change)| > 1, significant *P* < 0.05.

#### GO and KEGG enrichment analysis

We mapped all the genes to Terms in the Gene Ontology database and calculated the numbers of differentially enriched genes in each Term. Using top GOs to perform GO enrichment analysis (Ref to source data table: code. GO_KEGG) on the differential genes Calculate *P* by (the standard of significant enrichment is *P* < 0.05, *P* was calculated by hypergeometric distribution method) was used to find the GO term for significantly enriched differential genes to determine the main biological functions performed by differential genes. Package ggplot2 in R (Version 4.2.1) was used to draw a bubble diagram of the KEGG pathways participated by significantly differentially expressed genes, and we focused on the pathways with *P* < 0.05.

### Statistical analysis

Data were expressed as mean ± SEM. GraphPad Prism version 9.0 (GraphPad Software, USA) and R 3.4.3 were used for the statistical analysis. The Student’s test was used for comparisons between groups. One-way analysis of variance (ANOVA) was used for comparison among the three groups, and Tukey’s testing was then performed to identify statistical differences between the groups. *P* value < 0.05 was considered statistically significant.

## RESUILS

### Butyrate and its bacterial producers reduced in the gut of MG patients

**Fig. 1:**
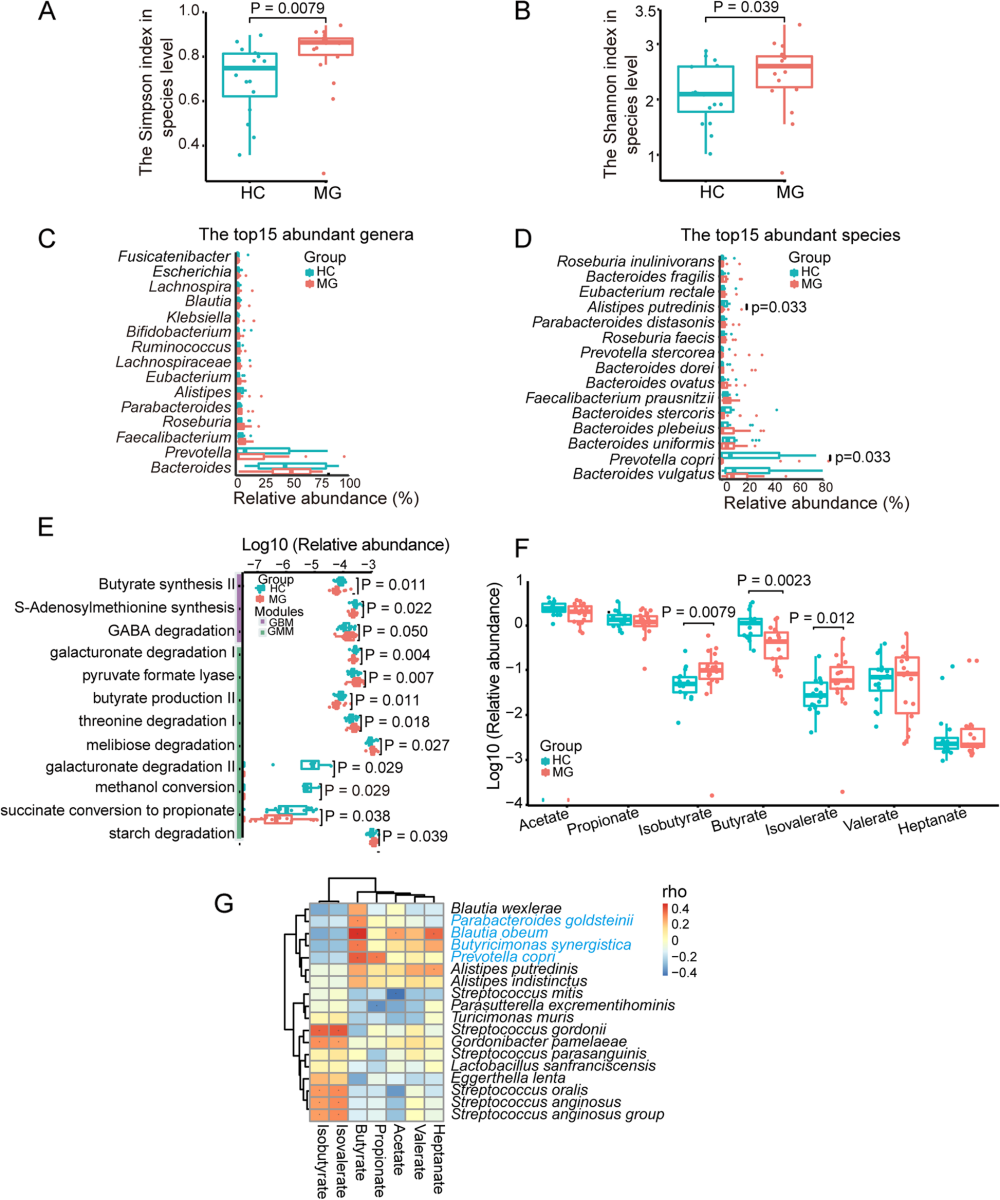
The characterization of the gut microbiome and SCFAs-related alterations in MG patients and healthy controls (HCs). **A** The Simpson index and **B** Shannon index at the species level. **C** The top 15 abundant genera and **D** the top 15 abundant species in the gut for MG patients and HCs. **E** The functional changes of the gut microbiota for MG patients and HCs. **F** Differences of levels of fecal SCFAs in MG patients and controls. **G** Spearman’s correlation analysis for SCFAs and significant discordantly abundant species (Wilcoxon rank-sum test), species marked in blue were enriched in HCs, while marked in black were enriched in MG patients. The blue boxes indicate negative correlations, while the orange boxes indicate positive correlations. *P < 0.05, *P < 0.01

Shotgun metagenomic data from 18 MG patients and 16 HCs (age and sex-matched) were used in the analysis. A total of 13 phyla, 132 genera, and 375 species were annotated by MetaPhlAn 3.0, significant differentially abundant phyla, genera, and species between MG patients and healthy controls (HCs) were shown in Table 1. The MG patients hold a higher diversity and richness of the species (Fig. 1A, Simpson index, *P* = 0.0079; Fig. 1B, Shannon index, *P* = 0.039). We have first focused on changes in abundant genera and species. For the top 5 abundant phyla, Proteobacteria (*P* = 0.027) was significantly abundant while Bacteroidetes (*P* = 0.036) was obviously lower in MG patients (Figure S1). The F/B ratio for HCs and MG patients was 0.24 and 0.36, respectively. No significant differences for the top 15 abundant genera were observed between MG patients and HCs (Fig. 1C). For the top 15 abundant species, the relative abundance of *Alistipes putredinis* and *Prevotella copri* were significantly lower in MGs (Fig. 1D). To further depict the changes in diversity and richness, we concentrated on the markedly different genera and species between MG patients and HCs (Table S2E, genera; Table S2F, species). Interestingly, *Butyricimonas* (*P* = 0.041), a butyrate producer in the gut, was significantly lower in MG patients, while *Eggerthella* (*P* = 0.010), *Parasutterella* (*P* = 0.027), *Gordonibacter* (*P* = 0.041), *Gemella* (*P* = 0.044), *Turicimonas* (P = 0.045) and *Enterococcus* (*P* = 0.049) were significantly higher in the MG patients. 18 markedly different species were analyzed from the MG patients and controls. It is worth noting that *Streptococcus* spp., including *S. oralis* (*P* = 0.005), *S. gordonii* (P = 0.009), *S. mitis* (*P* = 0.026), *S. anginosus* (*P* = 0.033), *S. parasanguinis* (*P* = 0.040) were all significantly increased in MG patients (Figure S2). Other species including *Eggerthella lenta* (*P* = 0.010), *Parasutterella excrementihominis* (*P* = 0.027), *Lactobacillus sanfranciscensis* (*P* = 0.034), *Gordonibacter pamelaeae* (*P* = 0.041), *Turicimonas muris* (*P* = 0.045) were obviously higher while *Blautia wexlerae* (*P* = 0.021), *Blautia obeum* (*P* = 0.038), *Butyricimonas synergistica* (*P* = 0.042), *Parabacteroides goldsteinii* (*P* = 0.043), and *Alistipes indistinctus* (*P* = 0.043) were evidently lower in MG patients.

**Table 1:** Significant differentially abundant phyla, genera, and species between MG patients and healthy controls (HCs)

	Items	*P* value	Mean abundance of HCs	Mean abundance of MG patients	Enrichment
**Phyla**	Bacteroidetes	0.0356	77.8074	68.8298	MG < HC
	Proteobacteria	0.0271	1.6230	4.6873	HC < MG
**Genera**	*Butyricimonas*	0.0407	0.1470	0.1432	MG < HC
	*Eggerthella*	0.0099	0.0035	0.0210	HC < MG
	*Parasutterella*	0.0271	0.3348	0.7212	HC < MG
	*Gordonibacter*	0.0406	0.0010	0.0096	HC < MG
	*Gemella*	0.0444	0.0012	0.0106	HC < MG
	*Turicimonas*	0.0449	0.0295	0.0518	HC < MG
	*Enterococcus*	0.0492	0.0021	0.0201	HC < MG
**Species**	*Prevotella copri*	0.0331	24.9951	10.6873	MG < HC
	*Alistipes putredinis*	0.0333	2.2294	1.3336	MG < HC
	*Blautia wexlerae*	0.0208	0.1290	0.0563	MG < HC
	*Blautia obeum*	0.0383	0.0954	0.0345	MG < HC
	*Butyricimonas synergistica*	0.0422	0.0558	0.0112	MG < HC
	*Parabacteroides goldsteinii*	0.0429	0.0441	0.0001	MG < HC
	*Alistipes indistinctus*	0.0432	0.0310	0.0085	MG < HC
	*Streptococcus oralis*	0.0054	0.0002	0.0043	HC < MG
	*Streptococcus gordonii*	0.0093	0.0001	0.0071	HC < MG
	*Streptococcus mitis*	0.0262	0.0021	0.0169	HC < MG
	*Streptococcus anginosus*	0.0325	0.0033	0.0137	HC < MG
	*Streptococcus parasanguinis*	0.0399	0.0225	0.0563	HC < MG
	*Eggerthella lenta*	0.0099	0.0035	0.0210	HC < MG
	*Parasutterella excrementihominis*	0.0271	0.3348	0.7212	HC < MG
	*Streptococcus anginosus group*	0.0325	0.0033	0.0137	HC < MG
	*Lactobacillus sanfranciscensis*	0.0339	0.0003	0.0243	HC < MG
	*Gordonibacter pamelaeae*	0.0406	0.0010	0.0096	HC < MG
	*Turicimonas muris*	0.0449	0.0295	0.0518	HC < MG

Gut-brain modules (GBMs) [38] and gut metabolic modules (GMMs) [41] were used to evaluate the function of the gut microbiota. Totally 43 GBMs were annotated, among which butyrate synthesis II (*P* = 0.011) and S-Adenosylmethionine (SAM) synthesis (*P* = 0.022) were significantly lower while GABA degradation (*P* = 0.050) was obviously higher in MG patients (Fig. 1E). 103 GMMs were obtained, in which butyrate production II (*P* = 0.011), galacturonate degradation I/II (*P* = 0.004, P = 0.029), methanol conversion (*P* = 0.029), succinate conversion to propionate (*P* = 0.038) were significantly decreased while pyruvate formate lyase (*P* = 0.007), threonine degradation I (*P* = 0.018), melibiose degradation (*P* = 0.027), and starch degradation (*P* = 0.039) were enriched in MG patients (Fig. 1E).

In summary, the low relative abundance of *Butyricimonas synergistica* and butyrate production modules in MG patients suggests a reduction in the levels of butyrate. GC-MS detection of the fecal SCFAs confirmed a decrease in the levels of butyrate (*P* = 0.0023) in MG patients (Fig. 1F). To further affirm the relationship between the markedly different species and SCFAs, spearman’s rank correlation analysis was performed and we found that *Blautia obeum*, *Prevotella copri*, *Butyricimonas synergistica*, *Parabacteroides goldsteinii* were all significantly associated with butyrate, whose relative abundance were all reduced in MG patients (Fig. 1G).

Collectively, the *Streptococcus* spp. were significantly increased while the relative abundance of SCFAs-producing species and butyrate production modules, and butyrate levels in the gut of MG patients were obviously decreased, suggesting butyrate supplementation might alleviate MG symptoms.

### NaB administration changed the gut microbial homeostasis in EAMG mice

**Fig. 2:**
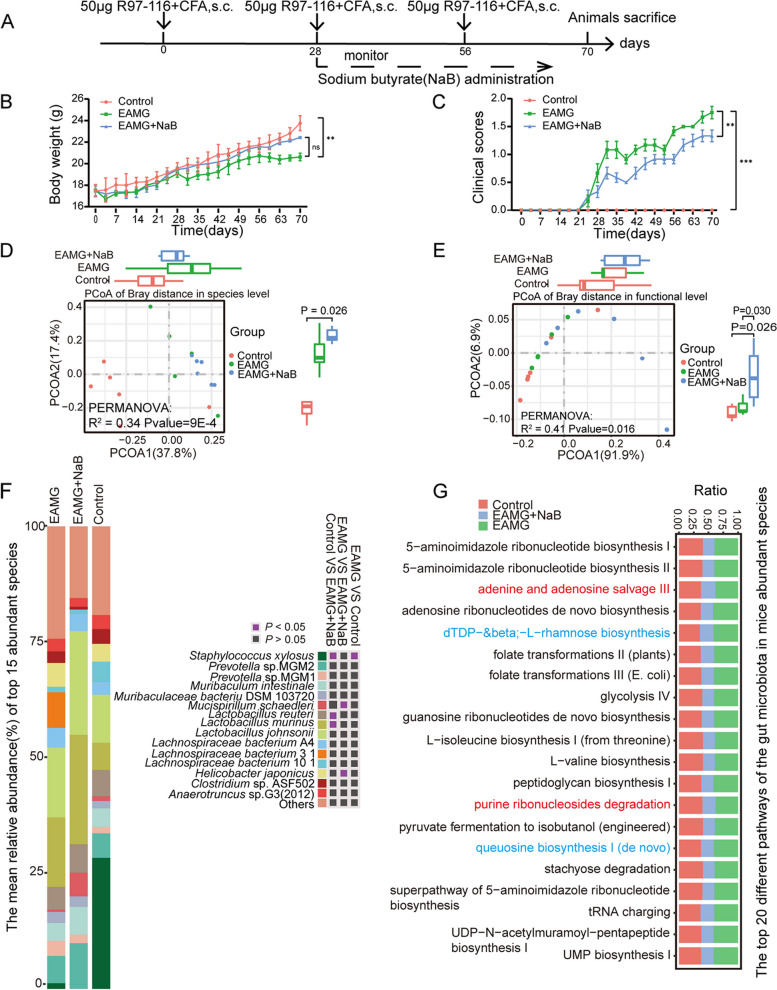
Compositional and functional changes in the gut microbiota of the EAMG mice. **A** Scheme of animal experiment. **B **Body weight. **C** Clinical scores. PCoA analysis of the gut microbiota at the (**D**) species level and **E** functional level. **F** The top 15 abundant species for three groups. **G** Top 20 different pathways for the gut microbiota among three groups. Data were from three independent experiments and expressed as mean ± SEM. The significance of differences was assessed by ANOVA, followed by Tukey’s testing as a post-hoc test. (n = 6 mice /group), ns means not significant, **p < 0.01 and ***p < 0.001

The EAMG mouse model was successfully constructed and NaB was gavaged to treat the EAMG mice for six weeks (Fig. 2A), and the MG symptoms were significantly improved (Fig. 2B and C) without organ destruction (Figure S3). The gut microbiota showed significant differences before and after treatment with NaB both at species (Fig. 2D) and functional level (Fig. 2E). The top abundant species were analyzed between EAMG mice and NaB-treated EAMG (MGD) mice (Fig. 2F). *Mucispirillum schaedleri* and *Helicobacter ganmani* were significantly reduced in MGD compared to the EAMG mice. Interestingly, species from *Lactobacillus* have no significance between the two groups except for *Lactobacillus murinus*. 6 phyla were annotated and among which Verrucomicrobia and Deferribacteres were increased in the MGD mice compared with EAMG mice and controls (Table S2L, Figure S4). 38 genera were annotated, and 11 genera were significantly different among the groups (Table S2M, Figure S5). Thereinto, *Parasutterella*, *Erysipelothrix*, *Proteobacteria*, and *Mucispirillum* were significantly higher while *Helicobacter*, *Adlercreutzia*, and *Asaccharobacter* were obviously lower in MGD mice compared with EAMG mice. Notably, *Akkermansia* was increased while *Bifidobacterium* was decreased in the MGD mice compared with EAMG mice. 64 species were totally annotated and 16 species were significantly different among the groups (Table S2N, Figure S6). *Parasutterella excrementihominis*, *Erysipelothrix larvae*, *Proteobacteria bacterium* CAG 139, and *Mucispirillum schaedleri* were significantly increased in MGD mice compared with EAMG mice while *Adlercreutzia equolifaciens*, *Asaccharobacter celatus*, and etc. were obviously reduced.

HUMAnN3 was used to exhibit the functional differences of gut microbiota and 278 pathways were obtained (Table S2O). Among these, 23 pathways including tryptophan biosynthesis, fatty acid elongation, and menaquinol biosynthesis were significantly decreased in the EAMG mice compared with controls. 98 pathways were significantly reduced in MGD mice compared with the EAMG mice, whose functions were mainly focused on amino acids biosynthesis, including L-isoleucine, L-valine, L-glutamate, L-glutamine, L-methionine, L-arginine, L-aspartate, L-asparagine, and L-proline, as well as L-histidine degradation, glycolysis, phosphatidylglycerol biosynthesis, adenosine, and guanosine biosynthesis. Only tRNA processing was obviously increased in MGD mice compared with EAMG mice. We have analyzed the top 20 abundant pathways (Fig. 2G) and found 16 pathways including adenosine ribonucleotides de novo biosynthesis, folate transformations III (E. coli), folate transformations II (plants), glycolysis IV, guanosine ribonucleotides de novo biosynthesis, peptidoglycan biosynthesis III (mycobacteria), stachyose degradation, tRNA charging, UDP-N-acetyl muramyl-pentapeptide biosynthesis I (meso-diaminopimelate containing), UMP biosynthesis I, 5-aminoimidazole ribonucleotide biosynthesis I, 5-aminoimidazole ribonucleotide biosynthesis II, L-isoleucine biosynthesis I (from threonine), L-valine biosynthesis, pyruvate fermentation to isobutanol (engineered), and super pathway of 5-aminoimidazole ribonucleotide biosynthesis were significantly reduced in NaB-treated EAMG mice compared with EAMG mice and controls. dTDP-&beta;-L-rhamnose biosynthesis and queuosine biosynthesis I (de novo) were obviously reduced in NaB-treated EAMG mice compared with EAMG mice. Adenine and adenosine salvage III and purine ribonucleosides degradation were significantly decreased in the NaB-treated EAMG mice compared with controls.

Our results revealed that NaB treatment could significantly change the composition and function of the gut microbiota in EAMG mice.

### NaB supplementation modulated the immune response in the EAMG mice

**Fig. 3:**
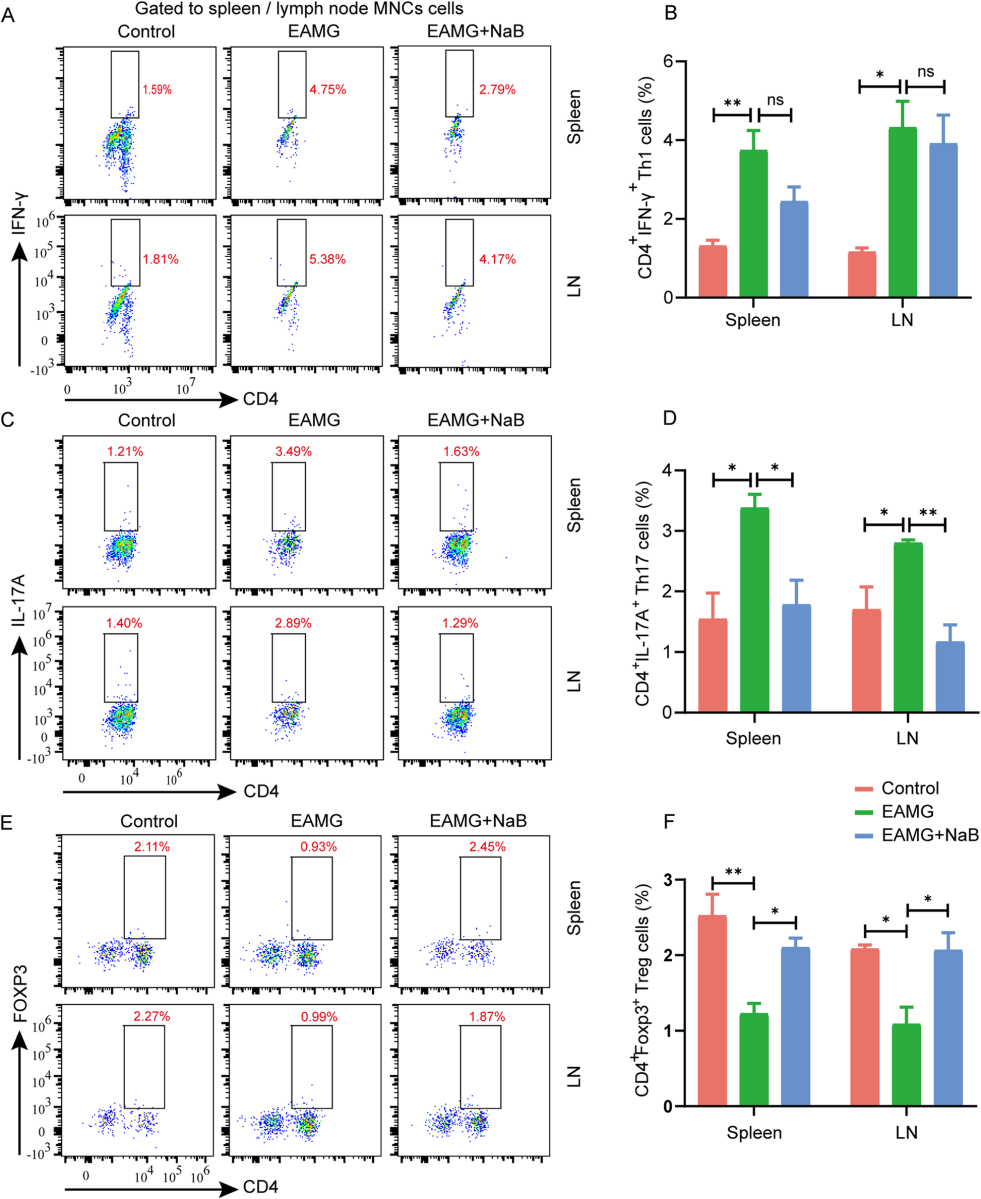
Effects of sodium butyrate on the T helper cell differentiation in EAMG mice. Mononuclear cells (MNCs) of the spleens and inguinal lymph nodes were isolated from mice in three groups on day 70. **A** Th1 cells, **C** Th17 cells, and **E** Treg cells were detected by flow cytometry. **B** The percentages of Th1, **D** Th17, and **F** Treg cells in MNCs were calculated. Data were from three independent experiments and expressed as mean ± SEM. The significance of differences was assessed by ANOVA, followed by Tukey’s testing as a post-hoc test. (n = 3 mice/group), ns means not significant, *p < 0.05 and **p < 0.01

**Fig. 4:**
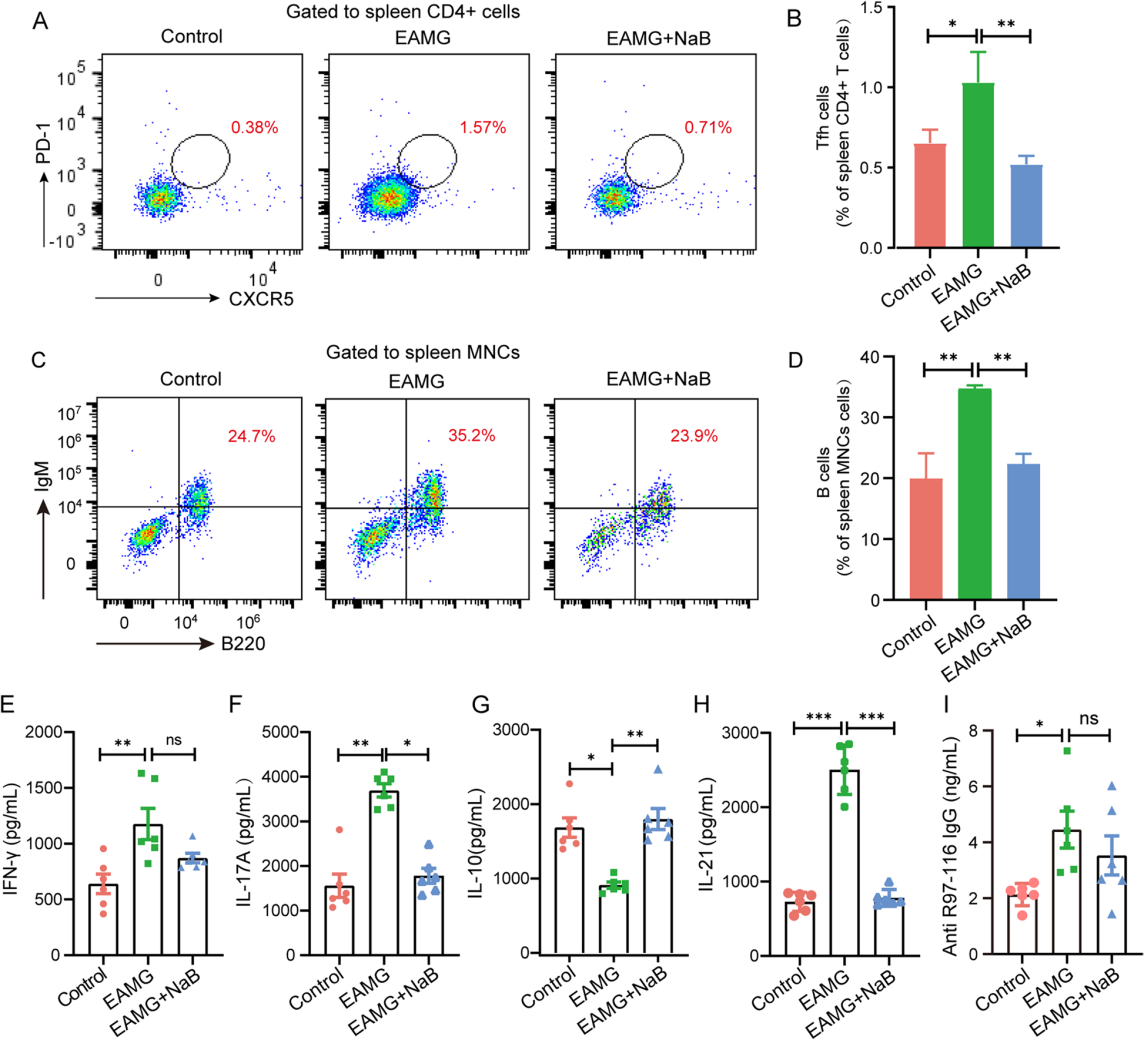
Effects of sodium butyrate on Tfh and B cell frequency and the representative cytokines and IgG antibodies levels. Mononuclear cells (MNCs) of the spleens were isolated from mice in three groups on day 70. **A** Tfh cells and (**C**) B cells were detected by flow cytometry. **B** The percentages of Tfh and **D** B cells in MNCs were calculated (n = 3 mice/group). Levels of **E** IFN-γ, **F** IL-17 A, **G** IL-10, **H** IL-21, and **I** titer of IgG antibody in the blood were measured by ELISA (n = 6 mice/group). Data were from three independent experiments and expressed as mean ± SEM. The significance of differences was assessed by ANOVA, followed by Tukey’s testing as a post-hoc test. ns means not significant, *p < 0.05, **p < 0.01, and ***p < 0.001

CD4 + T cell subsets are required for long-term antibody responses, and cytokines secreted mainly from CD4 + T cells regulate B cell antibody production [42]. The frequency of Th1 cells in EAMG mice exhibited a significant increase in comparison to the control group. However, after a 6-week treatment with NaB, the frequency of Th1 cells in EAMG mice decreased, although the difference was not statistically significant (Fig. 3A and B). Notably, alterations in IFN-γ levels across all three groups of mice corresponded consistently with changes in Th1 cells (Fig. 4E). However, an imbalance of Th17/Treg cells was observed, saying the number of Th17 cells was significantly increased while that of Treg cells was obviously decreased in the EAMG mice compared with controls. Interestingly, NaB supplementation for 6 weeks has dramatically reduced the numbers of Th17 cells (Fig. 3C and D) but increased that of Treg cells in the EAMG mice (Fig. 3E F). Accordingly, levels of IL-17 A were obviously increased while contents of IL-10 were evidently decreased in the EAMG mice compared with controls. However, NaB gavage for 6 weeks has significantly reduced the levels of IL-17 A (Fig. 4F) but increased the contents of IL-10 in the EAMG mice (Fig. 4G), suggesting NaB treatment could recover the R97-116 peptide-induced Th17/Treg imbalance in the EAMG mice. In addition, Tfh cells were also significantly increased in the EAMG mice but obviously decreased after being treated with NaB (Fig. 4A and B), and the changes in the level of IL-21 were consistent with the trend of Tfh change (Fig. 4H). Except for CD4 + T cells, the amounts of B cells and titers of anti-R97-116 IgG antibodies were also examined and the results showed that the numbers of B cells were significantly increased in the EAMG mice compared with controls but obviously decreased after being treated with NaB for 6 weeks (Fig. 4C and D). Interestingly, the titers of anti-R97-116 IgG antibodies were also significantly increased in EAMG mice but showed no significant changes after NaB treatment (Fig. 4I).

These results indicated that NaB administration could reverse the alterations in the numbers of CD4 + T cell subsets and B cell populations, as well as the corresponding cytokine levels in EAMG mice.

### Differentially expressed genes of B cells with and without NaB treatment

**Fig. 5:**
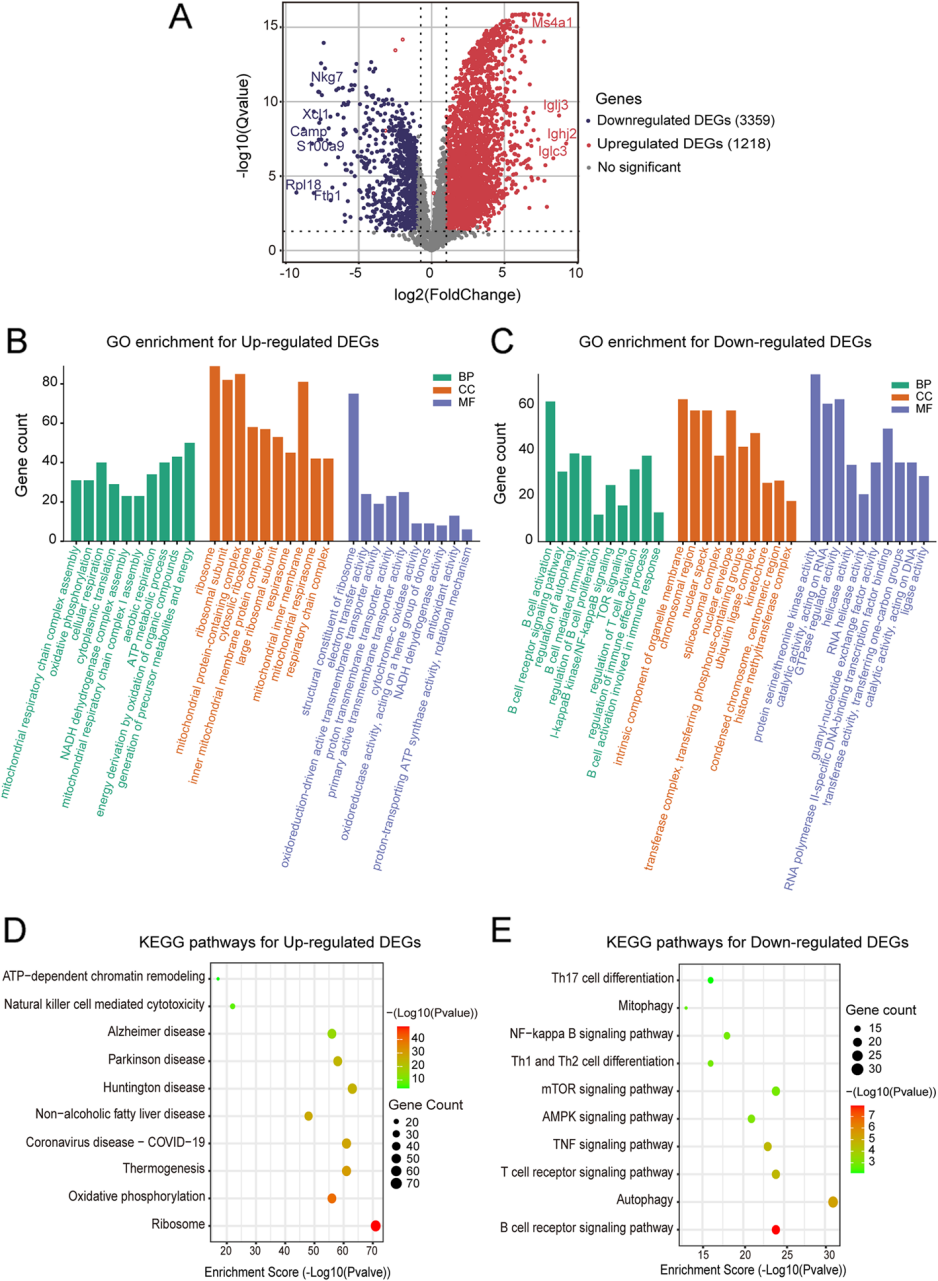
Transcriptomic sequencing and analysis of total B cells in spleen from mice in EAMG and NaB-treated group. **A** Volcano plot of differentially expressed genes (DEGs), in which 1218 DEGs were up-regulated while 3359 DEGs were down-regulated. GO enrichment at the biological process (BP), cellular component (CC), and molecular function (MF) for the up-regulated DEGs (**B**) and down-regulated DEGs (**C**) (The horizontal axis is the name of GO item). Filtered KEGG pathways related to MG or autoimmune diseases which annotated by the up-regulated DEGs (**D**) and down-regulated DEGs (**E**). (The bubble color changes from green-brown-red, the smaller the enrichment p Value, the greater the significance)

To further exhibit the function of B cells, RNA sequencing was performed on total B cells from different groups of mice. Totally, 4577 differentially expressed genes (DEGs) were detected between the EAMG mice and NaB-treated EAMG mice, in which 1218 DEGs were up-regulated and 3359 DEGs were down-regulated in the NaB treated-EAMG mice compared with EAMG mice (Fig. 5A, Table S2Q). GO annotation and KEGG pathway analysis were used to analyze the function of the up-/down-regulated genes. GO annotation **(**Fig. 5B and C) revealed that the down-regulation of DEGs primarily affects biological processes (BP) associated with B cell activation, autophagy regulation, and immune response. Conversely, the up-regulation of DEGs is primarily associated with the production of precursor metabolites and energy, as well as the energy generated through the oxidation of organic compounds. Regarding cell composition (CC), the down-regulation of DEGs mainly functions at the intrinsic component of organelle membrane, nuclear envelope, and chromosomal region. In contrast, the upregulation of DEGs was concentrated at the ribosome and mitochondrial protein-containing complex. As for the molecular function (MF), the up-regulated DEGs were associated with a structural constituent of ribosome, however, the down-regulated DEGs were associated with protein serine/threonine kinase activity and GTPase regulator activity. KEGG pathway analysis revealed that the up-regulated DEGs were mainly enriched in the ribosome, Huntington’s disease, Thermogenesis, and Coronavirus disease-COVID-19 (Fig. 5D), whereas the down-regulated DEGs were mainly enriched in immune and inflammatory-related pathways, such as autophagy, B cell receptor signaling pathway, T cell receptor signaling pathway, mammalian Target of Rapamycin (mTOR) signaling pathway, Tumor necrosis factor (TNF) signaling pathway, Th17 cell differentiation, Th1 and Th2 cell differentiation, Nuclear factor-kappa B (NF-κB) signaling pathway, mitophagy, and so on (Fig. 5E), suggesting that NaB treatment could reduce the expression of genes related to autoimmune and inflammatory responses in EAMG mice.

## Discussion

The role of gut microbiota and metabolites in MG has been a subject of investigation. This study aimed to examine the changes in gut microbiota and SCFAs in MG. The findings revealed a notable increase in gut microbial richness and diversity, alongside a decrease in butyrate levels. Furthermore, the impact and mechanisms of butyrate on MG were assessed through the construction of an EAMG model. The results demonstrated that butyrate significantly reduced the frequencies of Th17, Tfh, and B cells, as well as the levels of IL-17 and IL-21, while increasing the presence of Tregs and IL-10 in EAMG mice. Finally, the transcriptome of B cells from EAMG mice has verified the changes in biological processes and KEGG pathways related to inflammation and immune response.

As previously mentioned, we found that the gut microbial diversity and richness were significantly higher in MG patients, which might due to an increase in the *Streptococcus* spp. Qiu et al. demonstrated that compared to the healthy cohort, the gut microbial richness of the MG group was sharply reduced, particularly in the genus *Clostridium* [18]. This difference might come from (1) the average age of the cohort was 6 years older in our study; (2) MG patients in our study were in a period of disease while theirs were in a period of symptom stability; (3) the numbers of the cohort in our study were smaller than the published paper. Despite the inconsistent results of the gut microbial composition, our results of fecal SCFAs contents were consistent with the published studies. We have observed a significant reduction of the levels of SCFAs in the MG group, especially in butyrate, which might be due to a decrease in SCFA-producing bacteria including *Butyricimonas* [43], *Prevotella* [44], *Parabacteroides goldsteinii* [45], and *Alistipes indistinctus* [46] in MG patients. This result reminded us that butyrate supplementation for MG patients might be helpful to alleviate the symptoms of MG.

In order to investigate the impact of butyrate on myasthenia gravis (MG) symptoms, an experimental autoimmune MG model (EAMG) was developed by administering the R97-116 peptide at week 0 to induce immunity, followed by boosting immunizations at weeks 4 and 8. Sodium butyrate (NaB) was intragastrically administrated to the EAMG mice (once a day, 200 mg/kg/d) from weeks 4 to 10 during the second immunization on the 4th week. The MG symptoms were significantly alleviated in the NaB-treated mice compared with the EAMG mice. No progression was observed in the MG symptoms on the third immunization with R97-116 peptide at the 8th week in the NaB-treated group, but significantly higher clinical scores were observed in the EAMG mice. These results revealed that NaB gavage could relieve MG symptoms in EAMG mice.

Butyrate, an organic acid elicited by the gut microbiome, whose producer could thrive in a more acidic environment and in return, alter the pH of the intestinal microenvironment and thus affect the composition of the gut microbiota. In our study, we have revealed that species related to energy metabolisms, such as *Lactobacillus hominis* [47] and *Staphylococcus xylosus* [48] were significantly decreased. Meanwhile, the equol-producing bacterium *Asaccharobacter celatus* [49] was enriched in EAMG mice compared with controls, which suggested that the gut microbiota of EAMG mice has a poor capability of energy metabolism. All these species had fewer changes after being treated with NaB. In contrast, some beneficial species, including *Mucispirillum schaedleri* and *Parasutterella excrementihominis* were significantly enriched in the NaB-treated EAMG mice. *Mucispirillum schaedleri* could antagonize salmonella virulence to protect mice against colitis [50]. *Parasutterella excrementihominis* was an asaccharolytic and producer of succinate, which might have a potential role in bile acid maintenance and cholesterol metabolism [51]. We have also observed some bacteria including *Bacteroides dorei*, *Faecalibaculum rodentium et al.*, whose relative abundance was significantly decreased in the gut of NaB-treated EAMG mice. *Bacteroides dorei* was reported to be a novel immunobiotic ameliorating influenza virus infection in mice [52]. *Faecalibaculum rodentium* has an anti-tumourigenic effect with strong diagnostic, therapeutic, and translational potential [53]. *Helicobacter japonicum* was isolated from Japanese laboratory mice and can induce typhlocolitis and lower bowel carcinoma in C57BL/129 IL10−/− mice [54]. *Romboutsia ilealis* is adapted to a nutrient-rich environment where carbohydrates, amino acids, and vitamins are abundantly available [55]. *Asaccharobacter celatus* could not metabolize glucose or other carbohydrates as sole carbon sources [56]. *Adlercreutzia equolifaciens* was reported to metabolize isoflavones to equol, which has antioxidant properties greater than its parent isoflavone compounds [57]. These results suggested that the gut microbiota of the EAMG mice were significantly altered when treated with NaB.

Butyrate is an agonist for various GPCRs and stimulates anti-inflammatory cells including Tregs to reduce the activation of NF-κB and secretion of TNF-α to inhibit proinflammatory cytokines [58]. Activated T cells and cytokines play essential roles in the production of pathogenic autoantibodies and the induction of inflammation at the neuromuscular junction in MG [59]. Th1 cells strongly participate in the anti-AChR response and have a role in MG pathogenesis [6, 60]. However, no significant changes in the proportion for Th1 cells were observed after NaB intervention. Plentiful evidences have showed that Th17 cells could promote actication of B cell and could be therapeutic targets in autoimmune diseases [61]. IL-17, mainly secreted by Th17 cells, playing a pivotal role in the pathogenesis of MG, and IL-17 directed therapies may be a promising, targeted treatment for MG [62–65]. In our study, we revealed that NaB remarkably suppressed Th17 responses, as demonstrated by a reduced frequency of IL-17 producing CD4^+^ T cells. There is no consistent evidence in the alteration of the frequencies of Treg cells in AChR-MG [66–68]. The immune imbalance between Th17 and Treg cells contributes to the pathogenesis of MG [69]. NaB treatment could increase the frequencies of Treg cells, which is consistent with previous studies [70, 71]. Also, NaB treatment decreased the frequency of Tfh cells, which are capable of supporting somatic hypermutation B cells and antibody high-affinity maturation in germinal centers [72]. NaB would be expected to disrupting Tfh cells interactions with B cells by reducing Tfh cell frequencies, thereby reducing autoantibody production. These findings may provide preliminary support for butyrate as a potential alternative immunosuppressor for MG.

We have also examined the amounts of B cells and titers of IgG as MG is a B-cell-mediated autoimmune disease [1]. Our results revealed that NaB supplementation significantly reduced the number of B cells in EAMG mice. In addition, the transcriptome of B cells from spleen was conducted to elucidate the changes of gene expression and function of B cells with and without NaB treatment. Interestingly, the down-regulated DEGs in B cells of the NaB treatment group exhibited significant enrichment in various pathways, including mitophagy, B cell receptor signaling pathway, T cell receptor signaling pathway, mTOR signaling pathway, TNF signaling pathway, Th17 cell differentiation, Th1 and Th2 cell differentiation, and NF-κB signaling pathway, among others. The maintenance of mitochondrial integrity is critical for muscle health, mitophagy has been observed as selective autophagy invarious physiological processes and diseases, such as sarcopenia [73]. Moreover, mitophagy is closely related to the development, activation, and differentiation of T cells. Treg differentiation is dependent on mitochondrial lipid oxidation [74]. Mitophagy may be closely related to the function of CD4 + CD25 + Treg cells, which could explain the mechanism behind their effect on dysfunction in MG patients [75]. The mTOR signaling pathway is of utmost importance in the regulation of various cellular processes, including but not limited to cell proliferation, differentiation, apoptosis, and metabolism [76]. Additionally, it exerts its influence on the differentiation of Th17 cells through the regulation of hypoxia-inducible factor-1α, STAT3 phosphorylation, growth factor independent 1 downregulation, and RORγt transcription [77]. In their study, Lu et al. [78] demonstrated that the use of JAK2 inhibitors can effectively mitigate EAMG through the modulation of Th17/Treg balance via the inhibition of AKT/mTOR pathways and the reduction of IL-23 receptor expression. The primary role of TNF signaling pathway is in the regulation of immune cells. TNF-α is a potent proinflammatory cytokine located within the Human leukocyte antigen (HLA) locus that is tightly linked to the AH8.1 haplotype [79], which has been reported to be linked to early-onset MG in a Caucasian population [80]. NF-κB is the generic name of a family of transcription factors that function as dimers and regulate genes involved in immunity, inflammation and cell survival, dysregulation of NF-κB signaling, which is reported to be releted to MG pathophysiology [81], can lead to inflammatory, autoimmune disease and cancer [82]. Interestingly, NaB may indirectly affect the differentiation of Th1, Th2, and Th17 cells by affecting the gene expression of B cells. In all, the DEGs and invloved pathways identified in our study may provide insights into the molecular mechanisms of the differences between EAMG and NaB-treatd EAMG, which require further confirmation in future studies.

Our study has proved that the levels of butyrate were reduced in MG patients and NaB supplementation could significantly improve the MG symptoms in EAMG mice. We have not observed obesity in MG patients or obvious body weight loss in the NaB-treated EAMG mice although butyrate was reported to have the potential in alleviating obesity and related comorbidities [83]. The long effects of prolonged NaB administration on MG symptoms, body weight, blood lipids and glucose, and renal failure have not been evaluated in our study although butyrate has an effect on these aspects [84] since we have gavaged for 6 weeks of treatment and no weight loss and organs damage were observed in the course of our experiment.

## Conclusion

In conclusion, our study revealed an apparent reduction in the levels of butyrate and butyrate producing-bacteria in the gut of MG patients. Butyrate supplementation could significantly alleviate the symptoms of MG in EAMG mice by altering the gut microbiota, reversing the Th17/Treg cells imbalance, reducing the numbers of Tfh and B cells, and also regulating gene expression in B cells, which suggested the possible role of sodium butyrate in MG disease as a plausible treatment.

## Supplementary Information

**Additional file 1.**

## Data Availability

Metagenomic, metabolomics, and transcriptomic data supporting this study can be accessed at China National Genebank (https://db.cngb.org/cnsa/). Please refer to https://db.cngb.org/search/project/CNP0003937/.

## Abbreviations

MG: Myasthenia gravis
AChR: Acetylcholine receptor
MuSK: Muscle-specific kinase
LRP4: Lipoprotein-related protein 4
SCFAs: Short-chain fatty acids
NaB: Sodium butyrate
EAMG: Experimental autoimmune myasthenia gravis
DEGs: Differentially expressed genes
Tregs: Regulatory T cells
Th1/17: T helper 1/17
Tfh: T follicular helper
HCs: Healthy controls
QMGs: Quantitative Myasthenia Gravis score
HAMA: Hamilton Anxiety Scale
CFA: Complete Freund’s Adjuvant
ELISA: Enzyme Linked Immunosorbent Assay
H & E: Hematoxylin and eosin
GMMs: Gut metabolic modules
GBMs: Gut-brain modules
GC/MS: Gas chromatography/mass spectrometry
RNA-Seq: RNA sequencing
CC: Cellular component
MF: Molecular function
BP: Biological process
