# Correction: Sodium butyrate alleviates R97-116 peptide-induced myasthenia gravis in mice by improving the gut microbiota and modulating immune response

**DOI:** 10.1186/s12950-024-00408-8

**Published:** 2024-11-11

**Authors:** Jing Sun, Juanjuan Chen, Qinfang Xie, Mengjiao Sun, Wenjing Zhang, Hongxia Wang, Ning Liu, Qi Wang, Manxia Wang

**Affiliations:** 1 Department of Neurology, Lanzhou University Second Hospital, Lanzhou, 730030 China; 2 Cuiying Biomedical Research Center, Lanzhou University Second Hospital, Lanzhou, Gansu 730030 China; 3 Department of Neurology, Qinghai Provincial People’s Hospital, Xining, 810007 China

**Correction to: Sun et al. Journal of Inflammation (2023) 20:37**

10.1186/s12950-023-00363-w

After the publication of this article, it was reported that Fig. 3C & 4C contained errors. Specifically, the flow cytomet­ric dot plots were incorrectly overlaid on the gating line in Fig. 3C & 4C. This phenomenon can be attributed to the improper manipulation and adjustment of layers during the use of Adobe Illustrator software to collate the flow cytometry results. Such actions led to the misalignment of cells and gating lines, thereby diminishing the accuracy of the displayed results. The authors promptly submitted the original data to the journal’s editorial office for verifi­cation, accompanied by the corrected images. The original article has been updated by the authors.

The corrected figures are as follows:

Corrected Figure 3

**Fig. 3:**
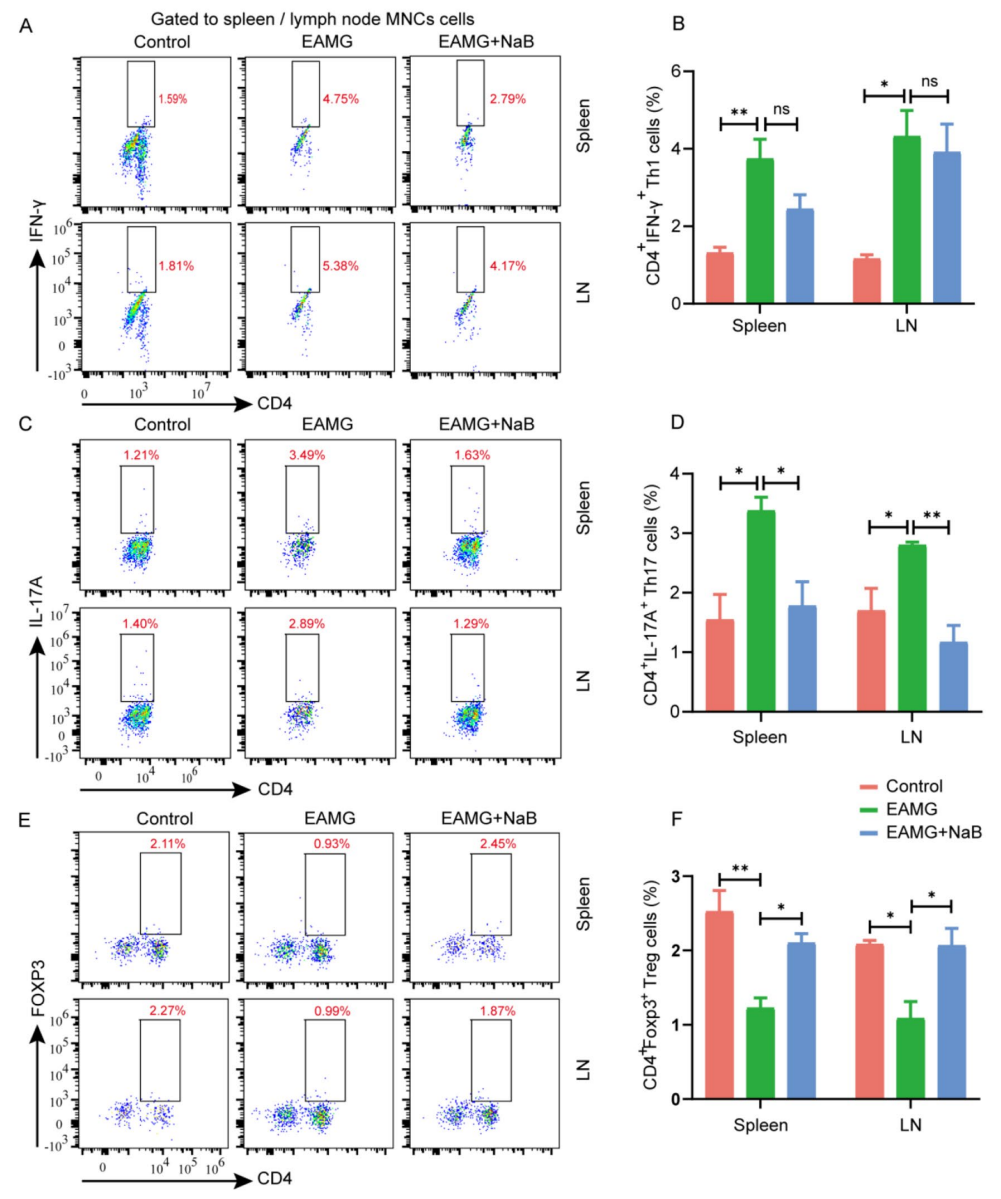
Effects of sodium butyrate on the T helper cell differentiation in EAMG mice. Mononuclear cells (MNCs) of the spleens and inguinal lymph nodes were isolated from mice in three groups on day 70. **A** Th1 cells, **C** Th17 cells, and **E** Treg cells were detected by flow cytometry. **B** The percentages of Th1, **D** Th17, and **F** Treg cells in MNCs were calculated. Data were from three independent experiments and expressed as mean±SEM. The significance of differences was assessed by ANOVA, followed by Tukey’s testing as a post-hoc test. (*n* = 3 mice/group), ns means not significant, *p < 0.005 and **p < 0.01

Corrected Figure 4

**Fig. 4:**
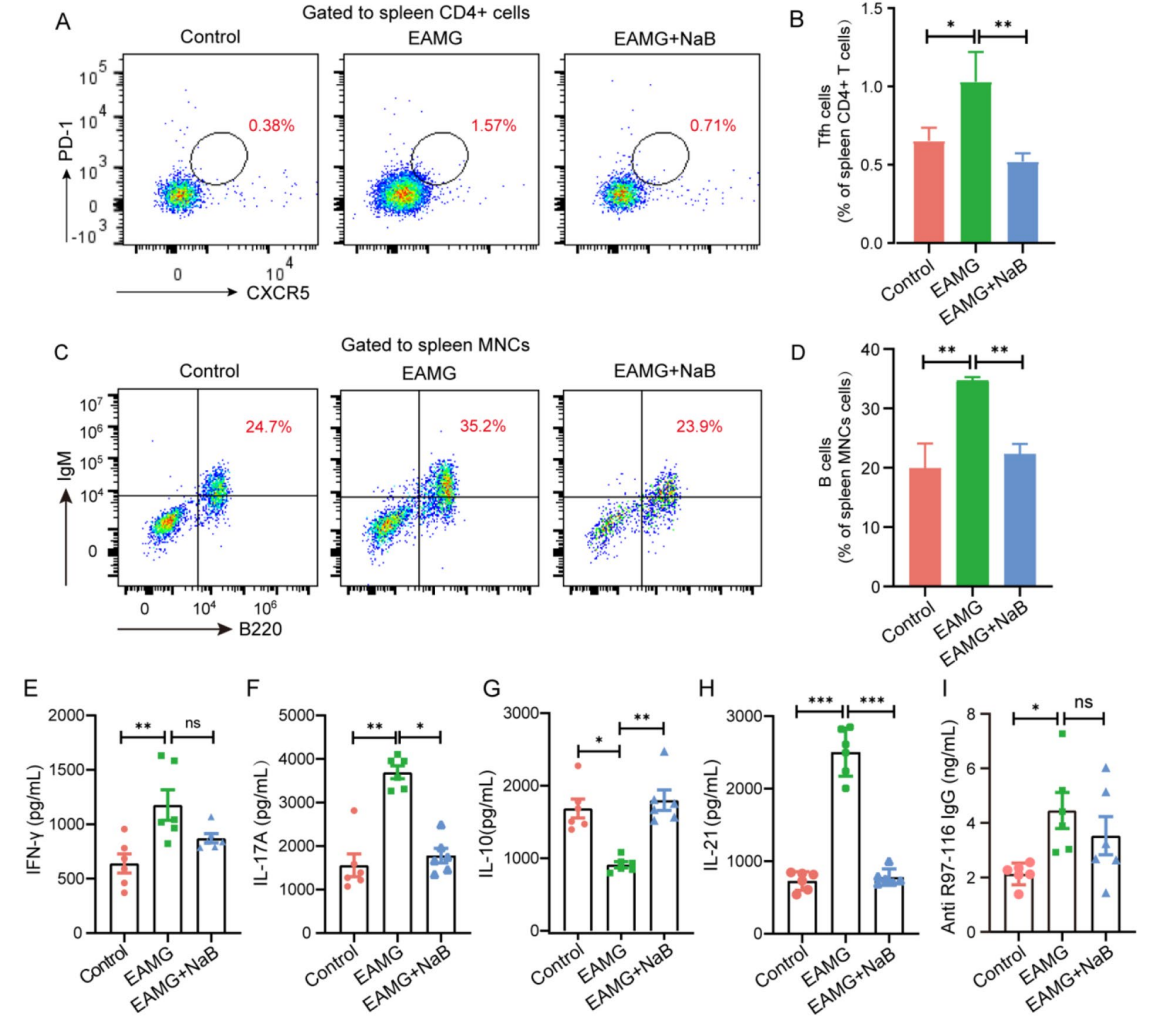
Efects of sodium butyrate on Tfh and B cell frequency and the representative cytokines and IgG antibodies levels. Mononuclear cells (MNCs) of the spleens were isolated from mice in three groups on day 70. **A** Tfh cells and **C** B cells were detected by fow cytometry. **B** The percentages of Tfh and **D** B cells in MNCs were calculated (*n*=3 mice/group). Levels of **E** IFN-γ, **F** IL-17 A, **G** IL-10, **H** IL-21, and **I** titer of IgG antibody in the blood were measured by ELISA (*n*=6 mice/group). Data were from three independent experiments and expressed as mean±SEM. The signifcance of diferences was assessed by ANOVA, followed by Tukey’s testing as a post-hoc test. ns means not signifcant, *p<0.05,**p<0.001

The Original Article has been corrected.

