# Supplementary material for: Sodium butyrate alleviates R97-116 peptide-induced myasthenia gravis in mice by improving the gut microbiota and modulating immune response
Source: J Inflamm (Lond). 2023 Nov 3;20:37. doi: 10.1186/s12950-023-00363-w (PMC10625296; doi:10.1186/s12950-023-00363-w)
Supplement: Supplementary file 1 — Additional file 1. [file 12950_2023_363_MOESM1_ESM.zip › Supplementary file/Supplementary Materials.docx]

Supplementary Material

## Sodium butyrate alleviates R97-116 peptide-induced myasthenia gravis in mice by improving the gut microbiota and modulating immune response

**Jing Sun^1#^, Juanjuan Chen^2#^, Qinfang Xie^1#^, Mengjiao Sun^1^, Wenjing Zhang^3^, Hongxia Wang^1^, Ning Liu^1^, Qi Wang^2*^, Manxia Wang^1*^***

**Corresponding authors:**

Qi Wang: [ery_wangqery@lzu.edu.cn](mailto:ery_wangqery@lzu.edu.cn);

Manxia Wang: [wmx32@aliyun.com](mailto:wmx32@aliyun.com)

# The supplementary materials include supplementary figures (Figure S1-S6) and tables. Table S1 was shown in the profile. Table S2A-Q was shown in Excel.

## Supplementary Figures

**Figure S1**. Relative abundance of all the phyla for MG patients and HCs.

**
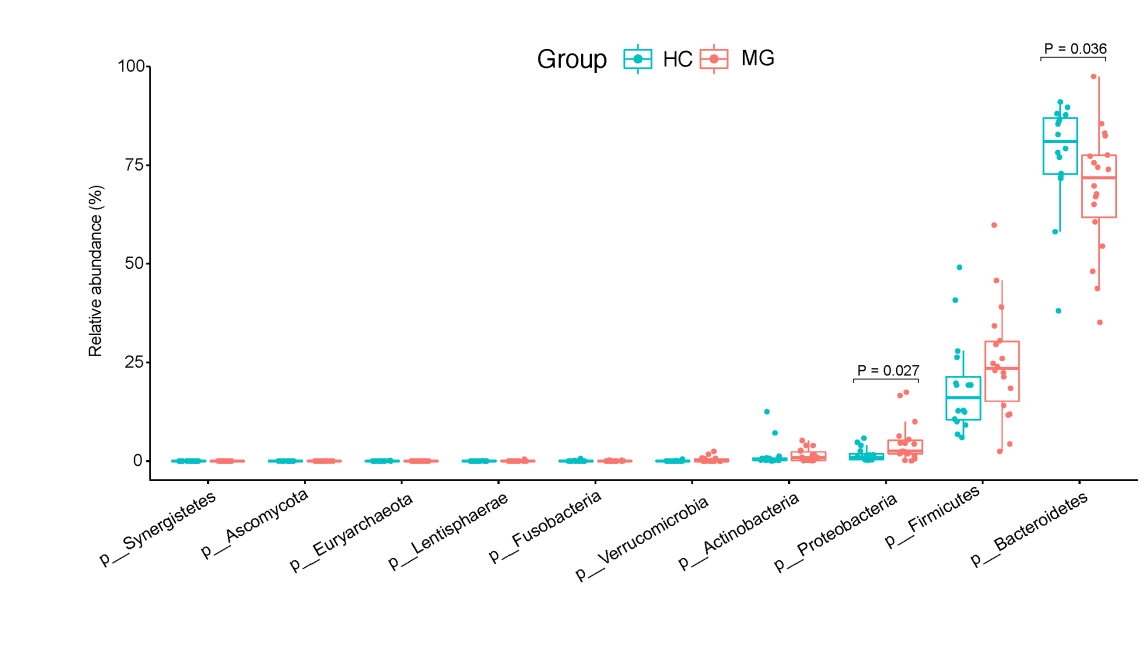
**

**Figure S2.** *Streptococcus* sp. was significantly abundant in MG patients.


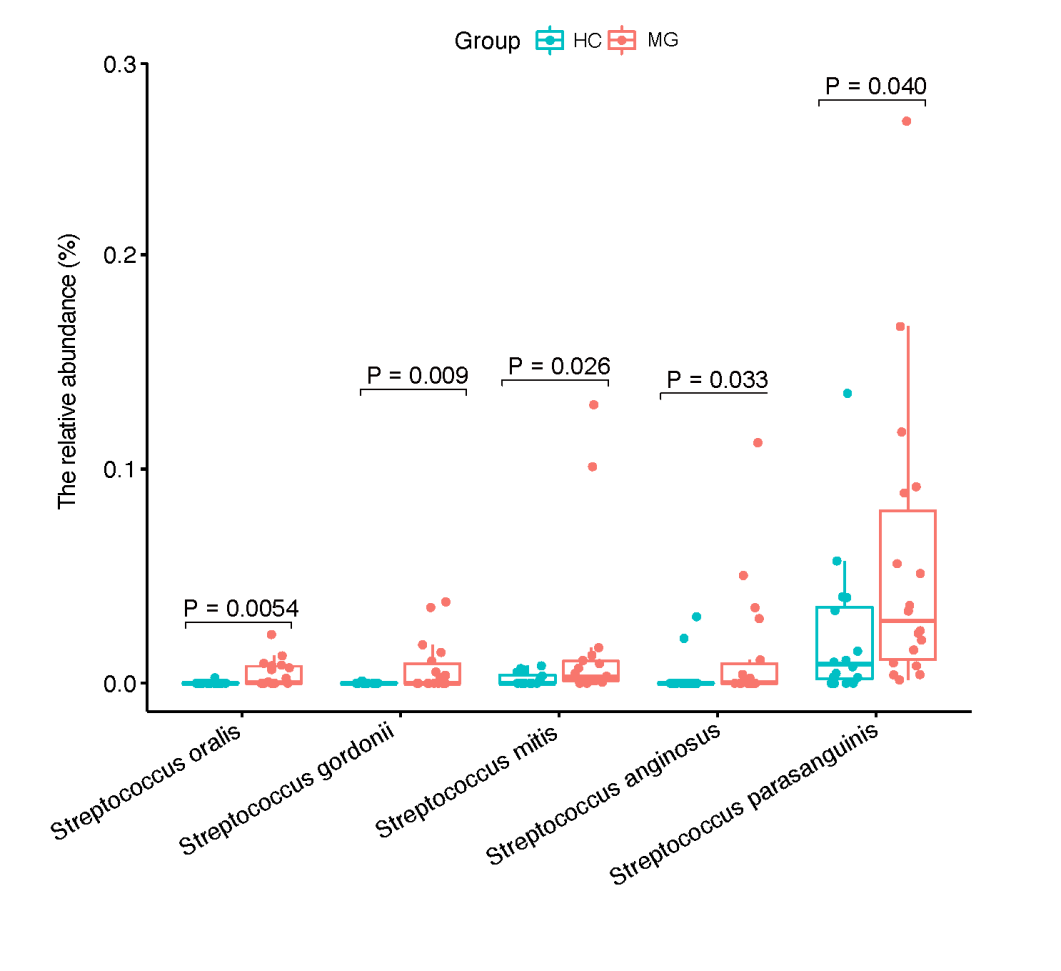


**Figure S3.** H&E staining of the heart, liver, spleen, lung, kidney, colon, and stomach of the mice in controls, EAMG group, and sodium butyrate-treated EAMG group showed no damage.


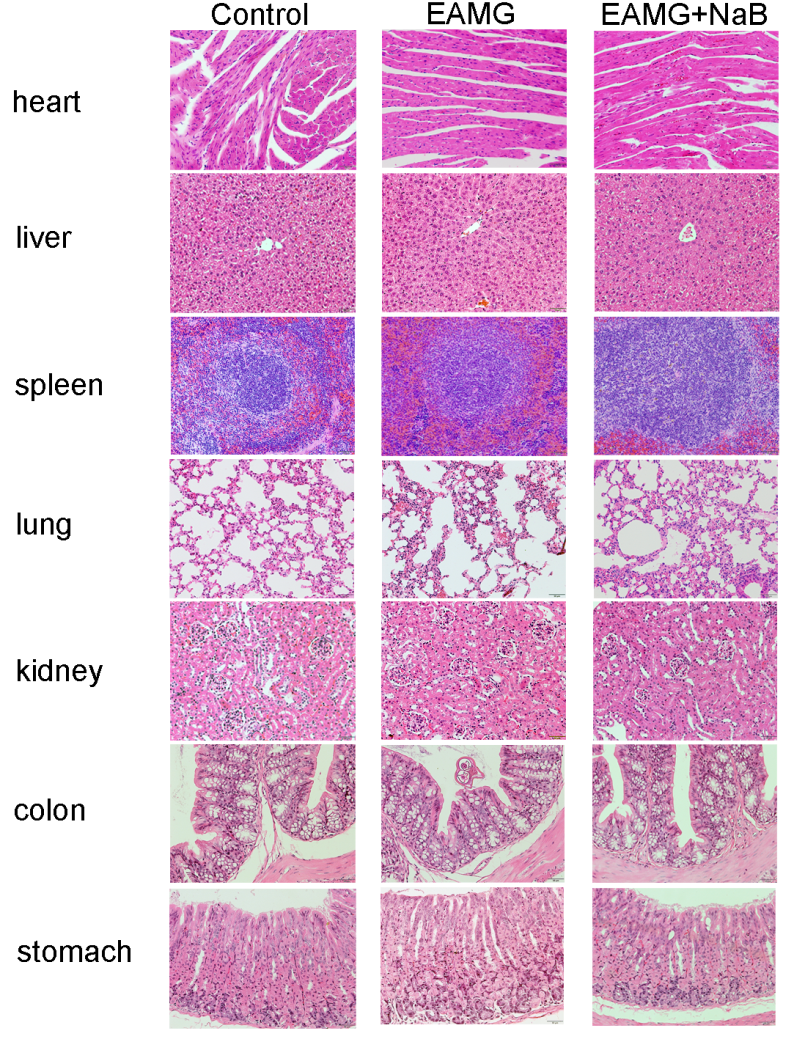


**Figure S4.** Significantly different phyla of the mice among the three groups. Control, controls; EAMG, EAMG mice; EAMG+NaB, sodium butyrate-treated mice.


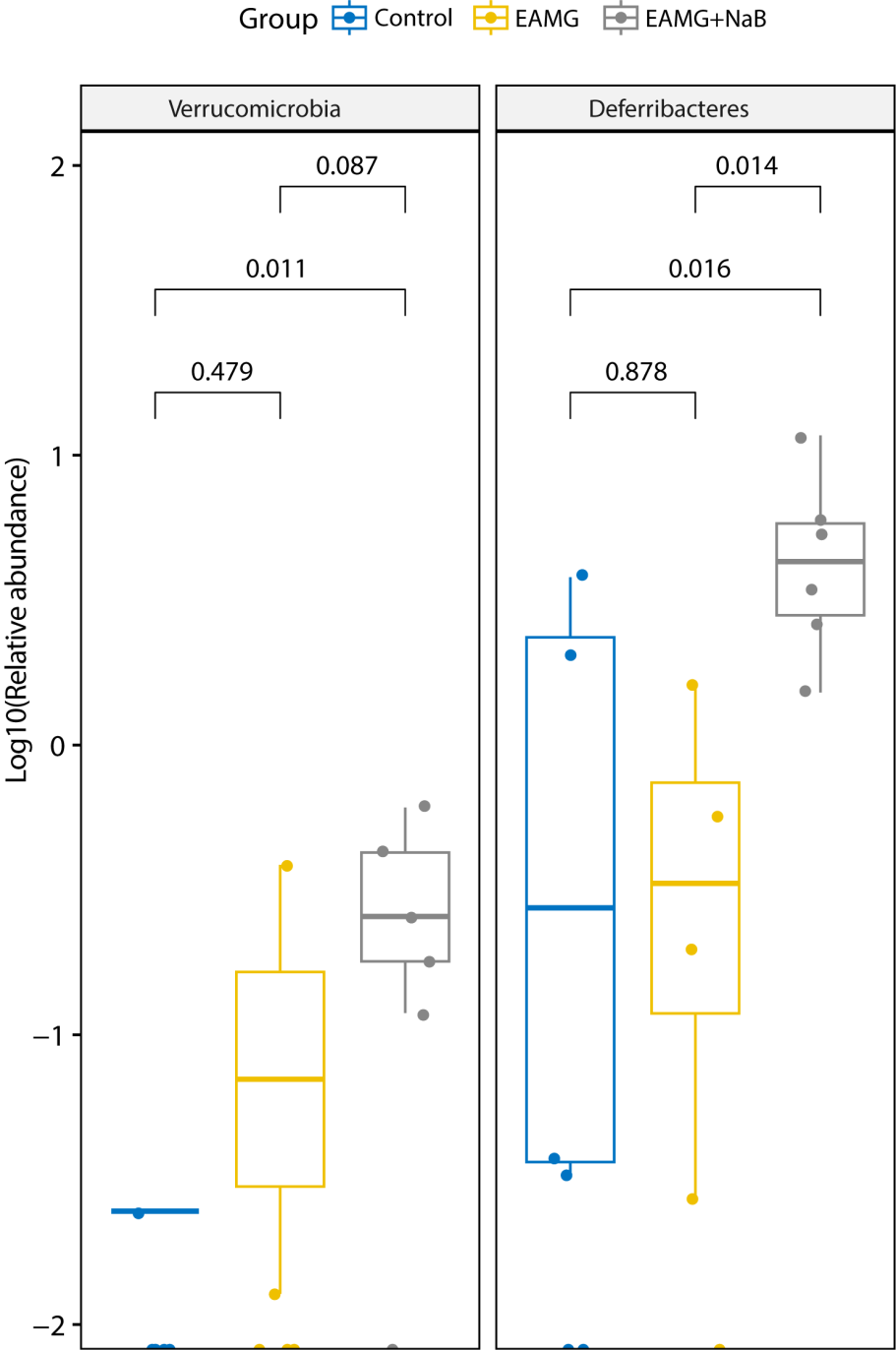


**Figure S5.** Significantly different genera of mice among the three groups. Control, controls; EAMG, EAMG mice; EAMG+NaB, sodium butyrate-treated mice.

**
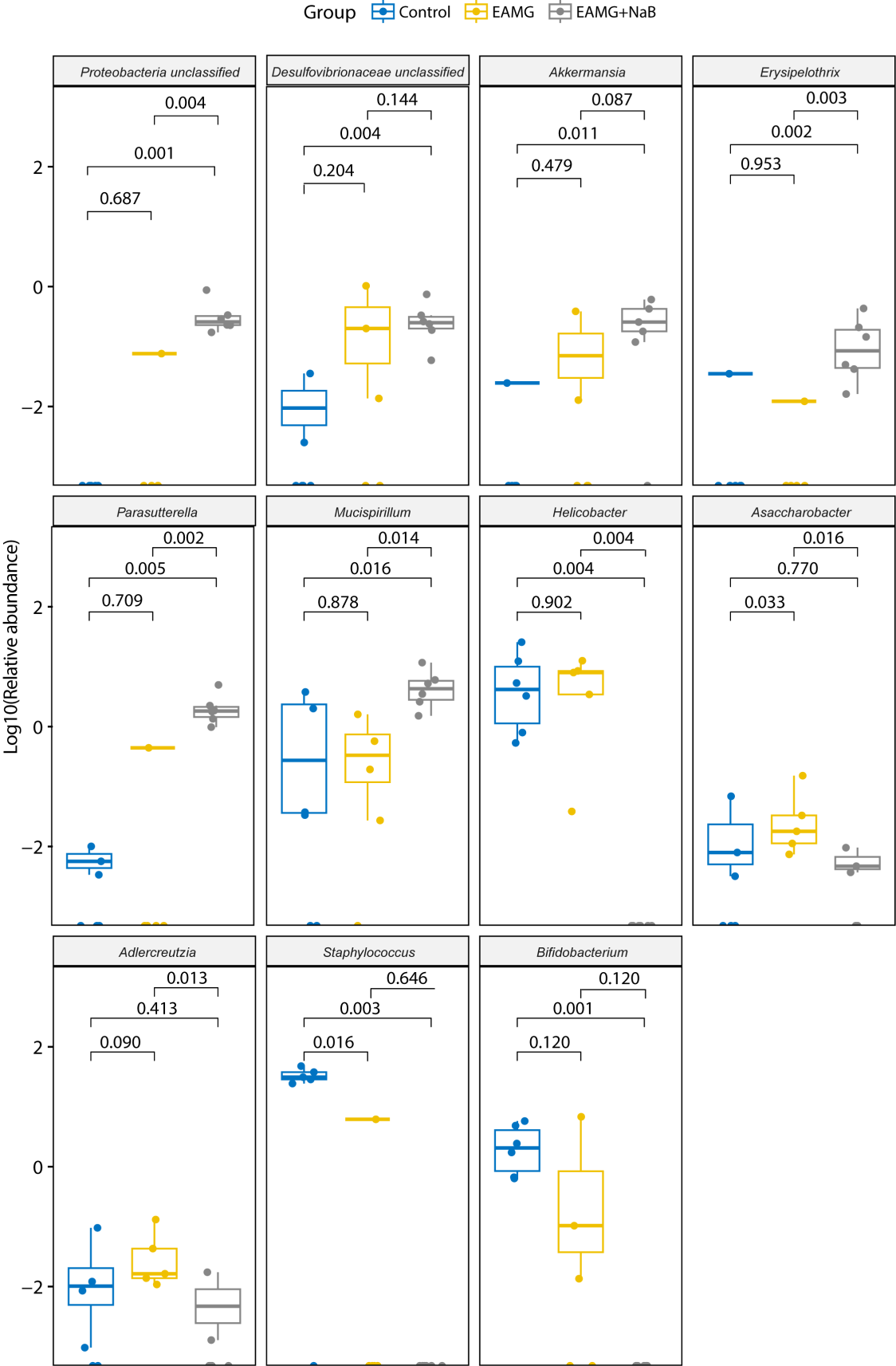
**

**Figure S6.** Significantly different species among the three groups. Control, controls; EAMG, EAMG mice; EAMG+NaB, sodium butyrate-treated mice.

**
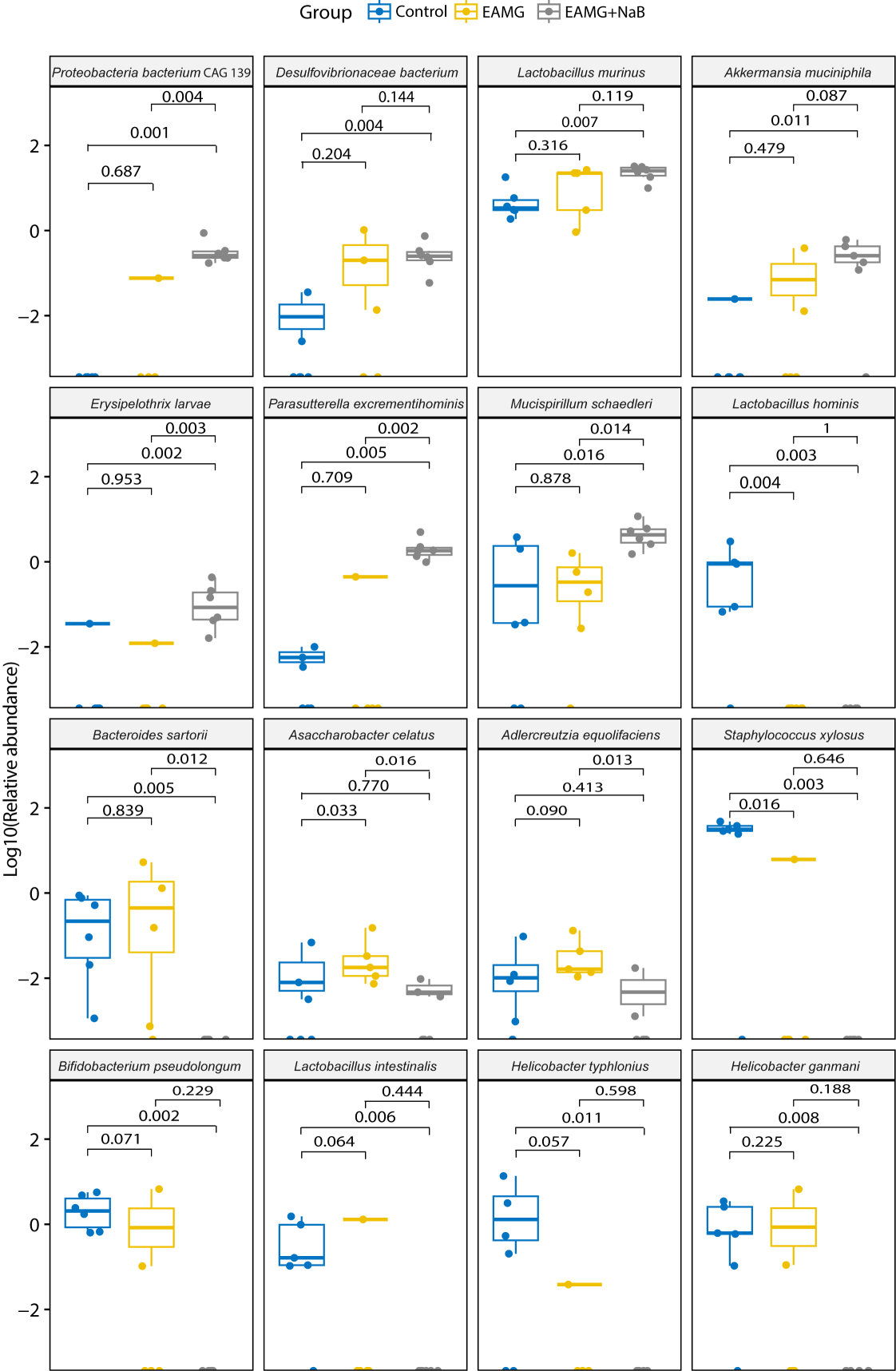
**

# Supplementary Tables

**Table S1**. Demographic and clinical characteristics of MG patients and healthy controls (HCs).

| Parameter | MG patients | HCs | *P* value^a)^ |
| --- | --- | --- | --- |
|  | n=18 | n=16 |  |
| Age, years^a)^ | 49.13±13.11 | 48.75±15.88 | 0.532 |
| Females, n (%) | 11(61.1%) | 10(62.5%) | 0.873 |
| Disease duration, month^b)^ | 19.93±35.24 | - | - |
| MGFA class, n% |  |  |  |
| I | 5(27.7%) | - | - |
| IIa | 3(16.7%) | - | - |
| IIb | 4(22.2%) | - | - |
| IIIa | 5(27.7%) | - | - |
| IVb | 1(5.6%) | - | - |
| Thymic abnormalities |  |  |  |
| Thymoma | 1(5.6%) | - | - |
| Thymic hyperplasia | 4(22.2%) | - | - |
| AChR Ab(+), n(%) | 30.03±28.97^b)^, 13（72%） | - | - |
| QMG score^c)^ | 18.31±7.13^b)^，13（72%） | - | - |

a)Two-tailed student test for continuous variables (age), Chi-square analyses for categorical variables (sex); b)Values are expressed as the mean ± standard error of the mean; c)Quantitative Myasthenia Gravis (QMG) test, QMG scores range from 0 to 39. Abbreviation: MG, myasthenia gravis.

**Table S2A**. Differentially enriched genera between MGs and HCs

**Table S2B**. Profiling of the gut microbiota in MG patients and controls.

**Table S2C**. Diversity of the gut microbiota in MG patients and controls.

**Table S2D**. Phyla differences in MG patients and controls.

**Table S2E**. Genera differences in MG patients and controls.

**Table S2F**. Genera differences in MG patients and controls.

**Table S2G**. Gut metabolic modules of the gut microbiota in MG patients and controls.

**Table S2H**. Gut-brain modules of the gut microbiota in MG patients and controls.

**Table S2I**. Fecal contents of SCFAs in MG patients and controls.

**Table S2J**. Profiling of the gut microbiota for mice.

**Table S2K**. Diversity of the gut microbiota.

**Table S2L**. Phyla of the gut microbiota.

**Table S2M**. Genera of the gut microbiota.

**Table S2N**. Species of the gut microbiota.

**Table S2O**. The function of gut microbiota in mice.

**Table S2P**. Humann 2 and species of gut microbiota in mice.

**Table S2Q**. Transcriptomics of the B cell in mice.
